# Curcumin Anti-Apoptotic Action in a Model of Intestinal Epithelial Inflammatory Damage

**DOI:** 10.3390/nu9060578

**Published:** 2017-06-06

**Authors:** Claudia Loganes, Sara Lega, Matteo Bramuzzo, Liza Vecchi Brumatti, Elisa Piscianz, Erica Valencic, Alberto Tommasini, Annalisa Marcuzzi

**Affiliations:** 1 Department of Paediatrics, Institute for Maternal and Child Health, IRCCS Burlo Garofolo, Via dell’Istria 65/1, Trieste 34137, Italy; claudia.loganes@gmail.com (C.L.); matteo.bramuzzo@burlo.trieste.it (M.B.); liza.vecchibrumatti@burlo.trieste.it (L.V.B.); erica.valencic@burlo.trieste.it (E.V.); alberto.tommasini@burlo.trieste.it (A.T.); 2 Department of Medicine, Surgery, and Health Sciences, University of Trieste, Strada di Fiume, 447, Trieste 34100, Italy; saralega83@gmail.com (S.L.); elisa.piscianz@burlo.trieste.it (E.P.)

**Keywords:** curcumin, epithelial cell line, intestinal inflammation, apoptosis, cytokines

## Abstract

The purpose of this study is to determine if a preventive treatment with curcumin can protect intestinal epithelial cells from inflammatory damage induced by IFNγ. To achieve this goal we have used a human intestinal epithelial cell line (HT29) treated with IFNγ to undergo apoptotic changes that can reproduce the damage of intestinal epithelia exposed to inflammatory cytokines. In this model, we measured the effect of curcumin (curcuminoid from *Curcuma Longa*) added as a pre-treatment at different time intervals before stimulation with IFNγ. Curcumin administration to HT29 culture before the inflammatory stimulus IFNγ reduced the cell apoptosis rate. This effect gradually declined with the reduction of the curcumin pre-incubation time. This anti-apoptotic action by curcumin pre-treatment was paralleled by a reduction of secreted IL7 in the HT29 culture media, while there was no relevant change in the other cytokine levels. Even though curcumin pre-administration did not impact the activation of the NF-κB pathway, a slight effect on the phosphorylation of proteins in this inflammatory signaling pathway was observed. In conclusion, curcumin pre-treatment can protect intestinal cells from inflammatory damage. These results can be the basis for studying the preventive role of curcumin in inflammatory bowel diseases.

## 1. Introduction

Chronic inflammation in inflammatory bowel disease (IBD) results from an inappropriate response to intestinal microbes in a genetically-susceptible host [1].

The incidence of IBD is increasing worldwide, and this increase seems to be more rapid in areas of the world that are experiencing a rapid socioeconomic transformation, particularly from low-income, rural to high-income, urbanized countries [2]. This epidemiological data, together with the observation that children of immigrants moving from countries with a low incidence of IBD to countries with high incidence have the same risk of developing IBD as the population of the country of immigration, indicate that environmental factors may play a major role in determining the risk of the disease [3].

Among the environmental factors, the role of diet gained a large amount of attention, and various substances in food have been found to influence the composition of gut microbiota, as well as the mucosal permeability and immune function within the gut [4]. Restrictive diets have a therapeutic role in pediatric Crohn’s disease (CD). Exclusive enteral nutrition (EEN) with elemental and semi-elemental formulas are the most widely studied restrictive diet which, in clinical trials in children with CD, proved to reduce clinical disease activity and improve mucosal inflammation [5].

The role of dietary supplements known for their anti-inflammatory properties such as vitamin D, soluble fibers, and long chain fatty acid have also been investigated both in animal models and clinical studies, and some evidence exists to suggest a potential protective role against the development or progression of gut inflammation [6]. 

Preventive dietary intervention may be critical, particularly in children, considered that there is a trend toward an earlier onset of IBD in Western countries. 

*Curcuma Longa*, also known as turmeric, is a perennial plant of the ginger family widely cultivated in Southern Asia. The root powder of *Curcuma Longa* is widely used for culinary purposes and in traditional medicine, mostly in Asia. The best-studied component of this plant is curcumin, which usually represents about 3% of turmeric powder. Curcumin has several pharmacological properties, including anti-inflammatory, anti-microbial, and anti-tumorigenic activities which seem all to be related to the ability of curcumin to modulate the expression of molecules involved in the inflammatory cascade and programmed cell death [7,8]. The role of curcumin in IBD has been investigated in two small, randomized, placebo-controlled trials where curcumin was given in association with a 5-aminosalicylic acid (5-ASA) compound to induce and maintain remission in patients with mild to moderate ulcerative colitis (UC). In both studies, the association of curcumin to 5-ASA was superior to the 5-ASA alone in inducing and maintaining clinical and endoscopic remission [9,10].

The effects of curcumin in gut inflammation has been studied both in mouse and human models providing a rationale for the possible beneficial role of curcumin use in IBD.

Preclinical studies in cellular and animal models showed that curcumin can modulate the inflammatory cytokine cascade and the vascular adhesion molecule expression and inhibit the nuclear factor-kappa B (NF-κB) [11]. Recently Midura-Kiela et al. found that, in human and mouse colonocytes, curcumin reduces the expression of IFNγ, one of the most prominent proinflammatory cytokines which drives a cascade of events that are thought to contribute to the pathogenesis of IBD [12].

In mouse models of colitis induced by chemicals, curcumin administration before the chemical insult exerted a protective effect limiting the histopathological damage and the activation of the inflammatory cascade [13,14,15]. 

In this study, we investigated the effect of curcumin pre-administration on human intestinal cells exposed to IFNγ as a proinflammatory insult.

We used the human colorectal adenocarcinoma cell line HT29. HT29 is a model of intestinal epithelium susceptible to the damage induced by inflammatory cytokines, such as IFNγ and TNFα which represent a suitable in vitro model to study the effect of inflammation on intestinal epithelia [16,17].

Increased apoptosis of epithelial cells is a well-known pathological feature in IBD. In particular, colonic biopsies of subjects with UC typically show increased apoptosis in crypt epithelial cells [18,19]; an increased enterocyte apoptosis is a common finding also in CD [20]. Of note, anti-inflammatory treatments also downregulate epithelial apoptosis [21]. This is particularly important if we consider that epithelial cell apoptosis can lead to barrier dysfunction and amplification of disease. Taken together, these data highlight the role of epithelial cells apoptosis in IBD. 

It is commonly assumed that curcumin exerts its action through antioxidant and anti-apoptotic effects [22,23,24]. However, other authors recently claimed that chemical properties of curcumin could have interfered with in vitro experiments in most studies, raising doubts on the beneficial effect of this substance [25]. To obtain more robust data, we analyzed the anti-apoptotic properties of curcumin by exploiting different methods including label-free measures. 

The primary aim of this study was to investigate whether pre-treatment with curcumin can prevent the development of apoptosis in HT29 cells treated with IFNγ. Apoptosis and cell death were measured by classical methods, such as flow cytometry, MTT assay, but also by more innovative label-free instrumentation measuring the change in impedance due to the adherent cells on the surface of the plate well.

In addition, we assessed if the action of curcumin on IFNγ-induced apoptosis is associated with a modulation of NF-κB signaling and inflammatory cytokine production [8,26,27].

We showed that pretreatment with curcumin 24 h before the administration of IFNγ can downregulate apoptosis in intestinal epithelial cells. Further studies will address whether curcumin anti-apoptotic properties may have a possible role in IBD prevention and treatment.

## 2. Materials and Methods

### 2.1. Cell Culture and Experimental Design

HT29 cells (human colorectal adenocarcinoma cell line), obtained from Sigma Aldrich (Sigma Aldrich, St. Louis, MO, USA) were cultured in Roswell Park Memorial Institute medium (RPMI) supplemented with 10% fetal bovine serum (FBS), 2 mM l-glutamine, 100 U/mL penicillin, and 0.1 mg/mL streptomycin (all from EuroClone, Milano, Italy). HT29 cells were seeded in different well plates, depending on the experimental needs, at a density of about 80,000–90,000/cm^2^.

**Figure 1:**
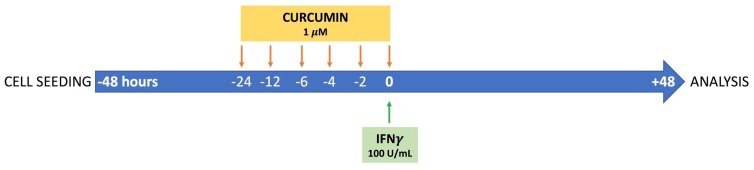
Representative illustration of the experimental design. Forty-eight hours after seeding, HT29 cells were stimulated with 100 U/mL IFNγ as inflammatory stimulus. To investigate the protective effect of curcumin, cells were pre-incubated with 1 µM curcumin (derived from *Curcuma Longa*) at different time points: 24, 12, 6, 4, 2, or 0 h before the IFNγ treatment. At 96 h of culture (48 h after the addition of IFNγ) cells were harvested for analysis.

Forty-eight hours after seeding, cells were stimulated with 100 U/mL IFNγ (Interferon Gamma 1b, Imukin, Boehringer Ingelheim, Milano, Italy) as inflammatory stimulus. To investigate the protective effect of curcumin, HT29 cells were pre-incubated with 1 µM curcumin (derived from *Curcuma Longa*, Sigma Aldrich) at different time intervals: 24, 12, 6, 4, 2, or 0 h before the IFNγ treatment. Curcumin alone, without any inflammatory stimulus, was also added to the cells to evaluate the effect on cell status. At 96 h of culture, after 48 h from the addition of IFNγ, cells were harvested for analysis. The experimental design is reproduced graphically in Figure 1. Curcumin was dissolved and solubilized in 100% ethanol (Sigma Aldrich). The final concentration of the vehicle in the cultured media did not exceed 0.01% (*v*/*v*).

### 2.2. Programmed Cell Death Assay

The apoptosis rate of HT29 was analyzed by flow cytometry using FITC-conjugated Annexin V (An) and propidium iodide staining (Annexin V-FITC Apoptosis Detection Kit, Immunostep, Salamanca, Spain), according to the producer’s instructions. HT29 cells were seeded in a 24-well plate at an initial concentration of 1.8 × 10^5^ per well. Forty-eight hours after IFNγ treatment, cells were harvested from the culture plate using 0.05% Trypsin-EDTA solution (EuroClone), washed with phosphate-buffered saline, and resuspended in the provided staining buffer for the incubation with Annexin V and propidium iodide. Flow cytometry analysis was performed on a BD FACSCalibur cytometer (Becton Dickinson, NJ, USA), and data were then analyzed with FlowJo software (version 7.6, Treestar, Inc., Ashland, OR, USA). The apoptotic cells (Annexin V positive, An+) were characterized based on the fluorescence emitted. Results were obtained by three independent experiments, performed in duplicate, and expressed as the fold increase after normalization on untreated controls.

### 2.3. Cell Viability Assay

Cytotoxicity was evaluated with MTT (3-(4,5-dimethylthiazol-2-yl)-2,5-diphenyltetrazolium bromide; Sigma Aldrich) colorimetric assay. Briefly, 2.5 × 10^4^ HT29 cells per well were seeded in a 96-well plate, pre-treated or not with curcumin for 24 h and stimulated with IFNγ at 48 h of culture. Ninety-six hours after seeding the MTT solution (5 mg/mL) was added to cell culture. The tetrazolium dye was reduced by metabolically-viable cells in purple formazan. After four hours of incubation at 37 °C, dimethyl sulfoxide was used to dissolve the insoluble formazan in a colored solution, quantified by a spectrophotometer at 595 nm (GloMax^®^-Multi+ Microplate Multimode Reader, Promega Corporation, Madison, WI, USA). The absorbance values are directly proportional to the number of viable cells and normalized to the untreated condition.

### 2.4. Analysis of Impedance with xCELLigence System

The xCELLigence RTCA DP Instrument (Roche, Penzberg, Germany) was used to assess number, viability, and morphology of the cultured cells. The xCELLigence RTCA system is a label-free approach that uses the electrical impedance of the adherent cells, expressed as a cell index (CI) value, to monitor the biological status of cells. With this instrument, any change in cellular morphology or growth rate that leads to detachment or death of cultured cells (for example the addition of toxic compounds) determines a reduction of the CI.

For this analysis, 2.5 × 10^4^ HT29 cells per well were seeded in a 16-well plate (E-plate; Roche) in 200 μL of complete medium, and cultured at 37 °C in 5% CO_2_. The cells were stimulated with IFNγ, with a curcumin 24-h pre-incubation. The impedance values were automatically measured every 15 min for 96 h. This assay was repeated twice in triplicate. The CI curves were normalized at the addition of IFNγ to compare more precisely the effect of the tested treatment. Ninety-six hours after seeding, CI was also normalized to the untreated control.

### 2.5. NF-kB Signaling Assay

The MILLIPLEX^®^ MAP 6-plex magnetic bead signaling kit (EMD Millipore Corporation, Billerica, MA, USA), was used to detect changes in phosphorylated NF-κB (Ser536), FADD (Ser194), IKKα/β (Ser177/Ser181), IκBα (Ser32) and in total protein levels of TNFR1 and c-Myc, in HT29 cell lysates. Briefly, cells were seeded in a 12-well plate at a cell density of 3.3 × 10^5^ per well, pre-incubated or not with curcumin, and then stimulated with IFNγ. Forty-eight hours after stimulation cell were harvested, washed with phosphate-buffered saline and lysed in the provided lysis buffer supplemented with protease inhibitors. To remove particulate matter, lysates were centrifuged at 14,000× *g* for 20 min at 4 °C. Cell lysates were analyzed following manufacturer’s instructions using the Luminex technology. Samples were acquired with the Bio-Plex^®^ 200 reader (BioRad, Hemel Hempstead, UK) and data were analyzed by Bio-Plex Manager^®^ software, which returned data as median fluorescence intensities (MFI). Results were represented as means and standard deviations of four replicate wells.

### 2.6. Cytokine Quantifications

Cytokines and chemokines released in the culture medium were quantified using the Bio-Plex Pro^®^ Human Cytokine 17-plex Assay, according to the manufacturer’s instructions (BioRad). Briefly, for the cytokine analysis, cells were seeded in a 12-well plate at a cell density of 3.3 × 10^5^ per well. Forty-eight hours after seeding, cells were pre-incubated or not with curcumin and then stimulated with 100 U/mL IFNγ. The cell supernatants were collected forty-eight hours after IFNγ treatment. Analytes examined by this method were Interleukin (IL)1β, IL2, IL4, IL5, IL6, IL7, IL8, IL10, IL12 (p70), IL13, IL17, granulocyte-colony stimulating factor (G-CSF), granulocyte/macrophage-colony stimulating factor (GM-CSF), interferon (IFN)γ, monocyte chemotactic and activating factor (MCP1; MCAF), macrophage inflammatory protein (MIP)1β, and Tumor necrosis factor (TNF)α. Data were acquired with a Bio-Plex^®^ 200 reader (BioRad), using Bio-Plex Manager^®^ software, which returned data as median fluorescence intensities (MFI) and concentrations (pg/mL).

### 2.7. Data Analysis

All results are expressed as means ± standard deviation (SD). Statistical significance was calculated using one-way analysis of variance (ANOVA) and Bonferroni post-test. Statistical significance was set at *p* < 0.05 (a), *p* < 0.01 (b), and *p* < 0.001 (c). Analyses were carried out using GraphPad Prism software (version 5.0, GraphPad Software Inc., La Jolla, CA, USA). 

## 3. Results

### 3.1. Time-Dependent Modulation of Apoptosis by Curcumin Pre-Treatment

**Figure 2:**
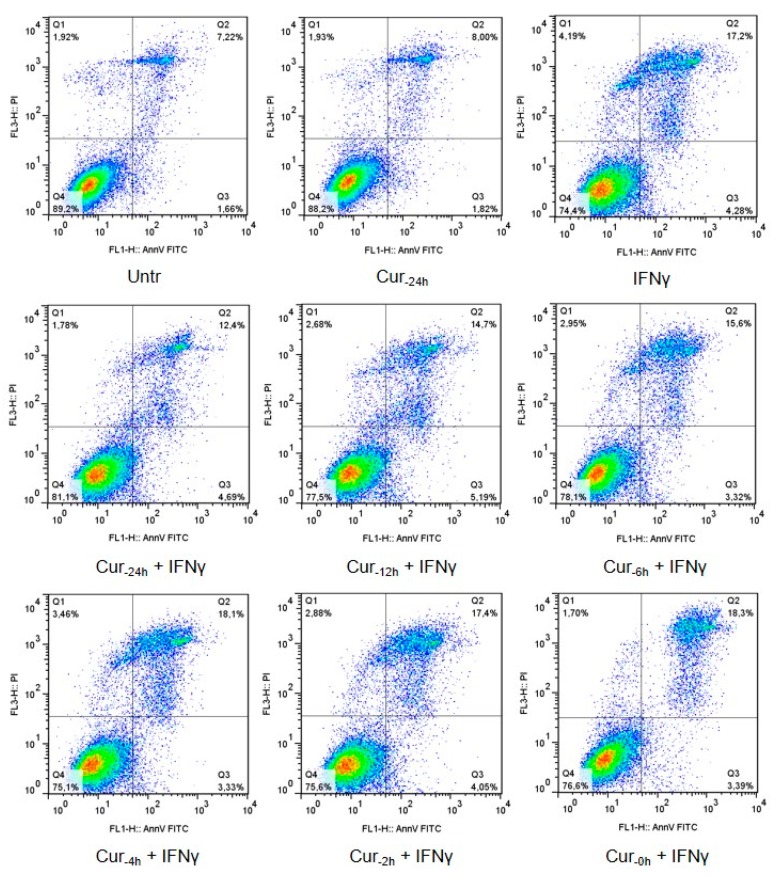
Representative flow cytograms of Annexin V-propidium iodide staining, for each experimental condition.

**Figure 3:**
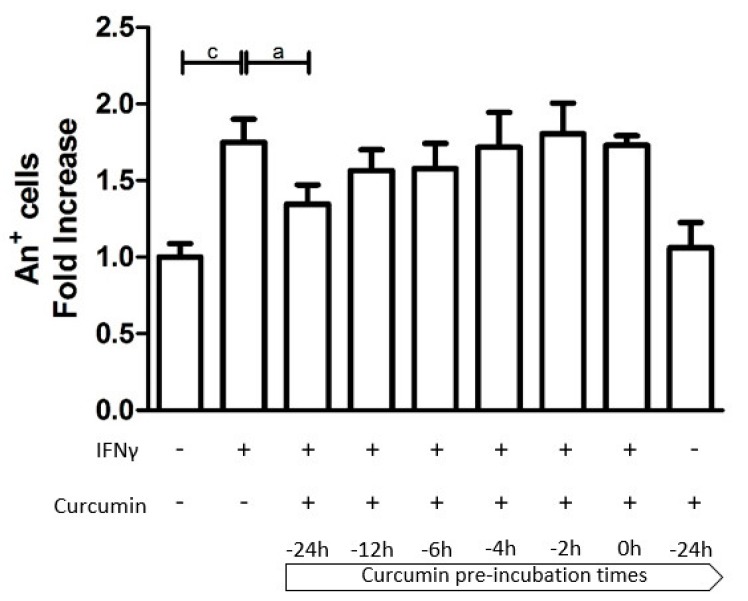
The effect of curcumin pre-treatment on HT29 apoptosis. The HT29 cell line was pre-incubated with curcumin at different time intervals and then stimulated with IFNγ for an additional 48 h. Programmed cell death is represented by Annexin V-positive cells (An+) expressed as a fold increase compared to untreated cells. Results are expressed as means ± SD of three independent experiments performed in duplicate. Data were analyzed with one-way ANOVA using the Bonferroni post-test (a: *p* < 0.05; c: *p* < 0.001).

Apoptosis rate of HT29 was assessed by the Annexin V-FITC (An) Apoptosis Detection Kit. As expected, treatment with IFNγ alone led to a significantly increased cell apoptosis (1.749-fold more An-positive cells compared with the unstimulated control, *p* < 0.001; Figure 2). Cultures pre-incubated with curcumin had a lower fraction of apoptotic cells compared to non-pre-treated cell cultures, with a trend of higher preventive effect at longer pre-incubation times. Indeed, the preventive effect was particularly evident, albeit not complete, when curcumin pretreatment was started 24 h before the addition of IFNγ (IFNγ: 1.749 vs. Cur_-24h_ + IFNγ: 1.348; *p* < 0.05). This beneficial effect gradually declined with the reduction of the pre-incubation period. Curcumin, alone (administered 24 h before the inflammatory stimulus), did not perturb the cell viability (Figure 2 and Figure 3).

### 3.2. Curcumin Effect on Cell Viability

**Figure 4:**
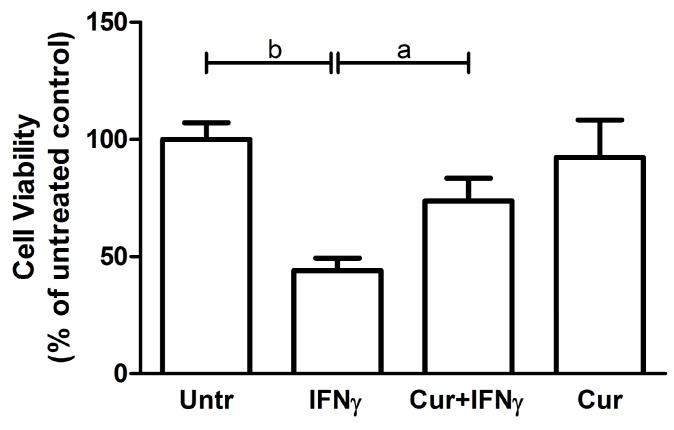
Viability analysis on HT29 pre-stimulated with curcumin. HT29 cell line was pre-incubated with curcumin (Cur) and after 24 h stimulated with IFNγ for an additional 48 h, to evaluate cell viability through MTT assay. Results are expressed as means ± SD, in percentage (%), normalized to the control condition (Untr). Data were analyzed with one-way ANOVA using the Bonferroni post-test (a: *p* < 0.05; b: *p* < 0.01).

The HT29 viability was evaluated with the MTT reagent, which allows quantifying the colorimetric reaction of the tetrazolium reduction to formazan in live cells. As expected, in the presence of IFNγ, the HT29 were metabolically less vital when compared to the untreated condition (Untreated: 100 ± 7.21; IFNγ: 44 ± 5.29; *p* < 0.01; Figure 4). Curcumin pre-incubation led to a significant, albeit partial, restoration of the metabolic activity, coherently with results from An V staining (Cur_-24h_ + IFNγ: 73.67 ± 9.81; *p* < 0.05).

### 3.3. Curcumin Effect on the Impedance Profile

**Figure 5:**
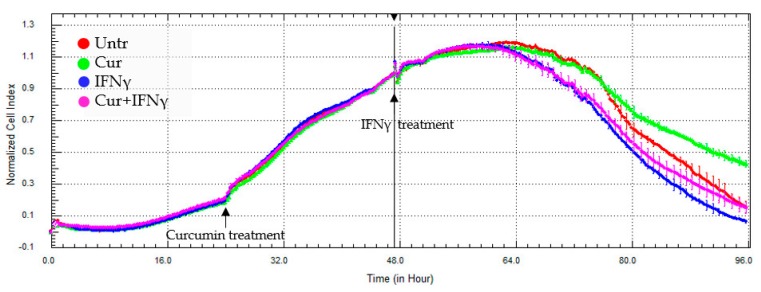
Representative impedance profile after curcumin pre-treatment. HT29 cells, cultured in a 16-well E-plate, were pre-treated with curcumin (Cur, 24 h after seeding), and stimulated with IFNγ (48 h after seeding) for a further 48 h. The graph shows the cell index values, normalized at the time of the IFNγ stimulation. The experiment was performed in triplicate.

**Figure 6:**
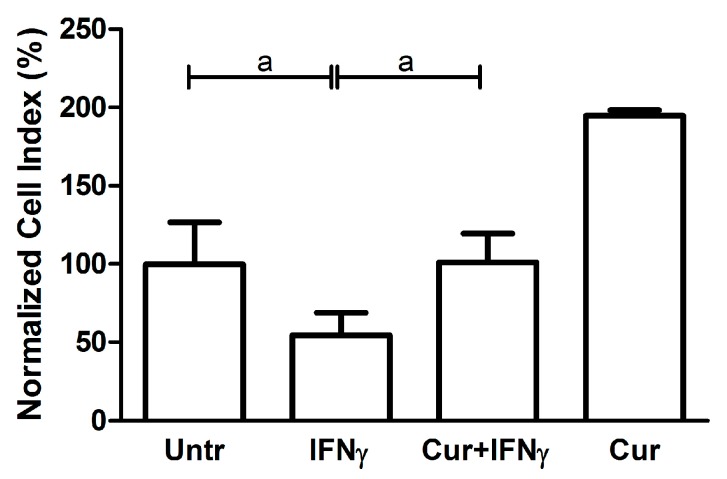
Impedance analysis of HT29 pre-treated with curcumin. The HT29 cells were pre-treated with curcumin (Cur, 24 h after seeding), and stimulated with IFNγ (48 h after seeding). The histograms represent, as a percentage (%), the mean of the normalized cell index values, reached after about 48 h IFNγ stimulation, compared to the untreated condition (Untr). Data are expressed as the mean ± SD. Data analyses were performed with one-way ANOVA and the Bonferroni post-test (a: *p* < 0.05).

The impedance measurement has confirmed the toxic effect of the IFNγ on cell status and the protective action of the curcumin administered 24 h before the addition of IFNγ. The representative xCELLigence graph (Figure 5) showed that the IFNγ addition led to a slight decrease in CI values (blue line), and the pre-treatment with curcumin stabilized the course of the line (pink line) up to the untreated condition (red line). Curcumin, alone, induced an increase of the impedance profile, possibly due to a change in cell morphology or adhesion strength (green line). Reporting the average of the normalized CI values obtained by different experiments after about 96 h of cell culture, it was possible to highlight that these differences are significant (Untr: 99.67 ± 26.84 vs. IFNγ 54.50 ± 14.39, *p* < 0.05; Cur_-24h_ + IFNγ: 101.0 ± 18.40, *p* < 0.05; Figure 6). 

### 3.4. Absence of NF-κB Modulation by Curcumin Pre-Treatment

**Figure 7:**
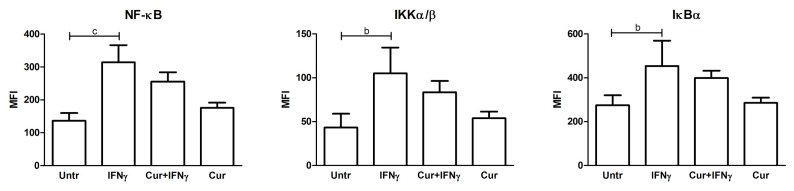
Multiplex analysis of phosphorylated proteins of NF-κB pathway in curcumin pre-treated HT29 cells. HT29 cells were pre-treated with curcumin (Cur) and then, after 24 h, stimulated with IFNγ for a further 48 h to detect changes in phosphorylated proteins belonging to the NF-κB pathway (NF-κB, IKKα/β, and IκBα), using a magnetic bead signaling kit and a Bio-Plex multiplex reader. Data, expressed as the median fluorescence intensity (MFI), represent the mean ± SD of four replicates and were analyzed with one-way ANOVA using the Bonferroni post-test (b: *p* < 0.01; c: *p* < 0.001).

IFNγ, as an inflammatory stimulus, was capable of activating the NF-κB (nuclear factor kappa B) pathway, as evidenced by the increased level of phosphoprotein of this signaling cascade, such as the transcription factor NF-κB and the upstream factors, IκBα and IKKα/β (NF-κB: 136.4 ± 23.39 vs. 314.5 ± 52.14 *p* < 0.001; IKKα/β: 43.20 ± 15.79 vs. 105.0 ± 29.60 *p* < 0.01; IκBα: 274.8 ± 45.38 vs. 453.7 ± 116 *p* < 0.01). The pre-treatment with curcumin lowered the levels of these phosphorylated analytes slightly, but these trends were not significant (Figure 7). The other proteins analyzed (FADD, TNFR1, and c-Myc) did not show any significant differences after the inflammatory and curcumin treatment (data not shown).

### 3.5. Downregulation of the IL7 Secretion by Curcumin

**Figure 8:**
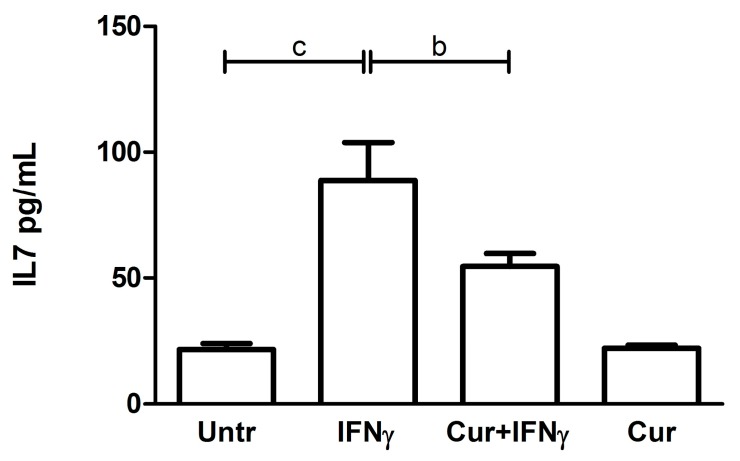
IL7 quantification after curcumin pre-treatment. The HT29 cell line was pre-incubated with curcumin (Cur) and, after 24 h, stimulated with IFNγ for an additional 48 h to evaluate the cytokine secretion. The histograms represent the concentration (pg/mL) of IL7 expressed as the mean ± SD of four replicates. Data were analyzed with one-way ANOVA using the Bonferroni post-test; (b: *p* < 0.01; c: *p* < 0.001).

The IFNγ treatment induced an increase of IL7 release in the HT29 culture media (Untr: 21.51 ± 2.40 vs. IFNγ: 88.76 ± 15.01, *p* < 0.001, Figure 8). When administered 24 h before the inflammatory stimulus, curcumin led to a significant downregulation of the levels of cytokines secreted, compared with the values obtained after the administration of IFNγ alone (IFNγ: 88.76 ± 15.01 vs. Cur_-24h_ + IFNγ: 54.54 ± 5.25, *p* < 0.01, Figure 8). With respect to the other analytes, no significant and biological differences were found between the four analyzed conditions (data not shown).

## 4. Discussion

Curcumin, the active substance of *Curcuma longa* which is attributed most of the beneficial properties of turmeric, is chemically classified among the polyphenols.

The many beneficial properties attributed to curcumin, as antioxidant, anti-inflammatory, anti-infective, anti-tumor, and preventive agent, are closely correlated with each other and could be associated with the ability to modulate the molecules involved in inflammation, programmed cell death, and morphology.

In the present work, we demonstrated that pre-treatment with curcumin is able to reduce the levels of apoptosis in intestinal epithelial cells exposed to IFNγ. The fact that concordant data were obtained with different methods strengthens the interpretation of the results. In particular, there was a recent warning about the possibility that much of the research data on curcumin may be weakened, or even invalidated, when natural properties of fluorescence and protein binding of the compound are taken into account [25]. The inclusion in our research plan of a label-free assay based on cell impedance analysis allows ruling out such interferences. 

When we further investigated the protective mechanisms of action of curcumin on HT29 cells, we observed that pre-treatment with this compound led to a significant reduction in the amount of IL7 secreted in response to IFNγ. IL7 is a crucial cytokine, usually secreted by intestinal epithelial cells in response to bacterial infections, which contributes to the organization of secondary lymphoid structures [28,29]. While basal secretion of this cytokine in response to the intestinal microbiota may be required for an optimal homeostasis of the mucosal immunity [30,31], higher levels may play a role in the pathogenesis of IBD and inhibition of IL7 has been proposed as a complementary approach to target inflammation in these disorders [32,33,34,35]. Since mucosal production of IL7 in vivo has been implicated in the activation of pathogenic CD4 T lymphocytes, the action of curcumin on this cytokine may be independently useful to contrast intestinal inflammation [36,37]. 

Although a cell line cultured in the laboratory can hardly reflect the complex physiology of intestinal mucosa, HT29 cells are considered a good model to study cytokine-induced apoptosis in epithelial cells. The model is coherent with in vivo data showing that epithelial apoptosis is increased in intestinal mucosa in subjects with the active disease while it is reversed by anti-inflammatory treatment with anti-TNFα antibodies. Increased apoptosis in epithelial cells is thought to contribute to increased permeability and amplification of inflammation. Thus, inhibiting inflammation-induced epithelial cell apoptosis may be relevant to the prevention or the treatment of IBD, as suggested by recent data [10]. 

It is not clear how curcumin acts on IFNγ-induced apoptosis and cytokine secretion. One of the known actions of curcumin regards iron chelation and reduction [38,39,40,41], but it is not clear if the anti-inflammatory and anti-apoptotic effects of the drug may depend on this property. We evaluated NF-κB activation since this pathway is a known target of IFNγ. Even if there was a trend toward reduction of the levels of phosphorylated components of NF-κB, this did not reach statistical significance. Thus, we could not conclude that the downregulation of the NF-κB pathway is the main mechanism of action of curcumin. A possible alternative explanation is that curcumin exerts its anti-inflammatory property by a direct modulation of mitochondrial function, as suggested by other authors [42]. 

Although we must put much caution in translating data obtained in vitro on a cell line, we can hypothesize that our results may be relevant to the understanding of the action of curcumin in vivo. In particular, a protective effect of curcumin was described in a mouse model of UC [43]. Moreover, small clinical trials in UC seem to suggest that curcumin may be a promising and safe complementary medication for maintaining remission in patients with quiescent disease [44].

An issue that should be addressed to assess the real potential of curcumin into the clinic concerns its poor aqueous solubility and low bioavailability, which may hinder in vivo efficacy [45]; low plasma levels and limited tissue distribution of curcumin appear to be due to poor absorption, high-rate metabolism, and rapid systemic clearance. A possible solution to these issues would be the development of different types of curcumin formulations, such as nanoparticles, liposomes, or phospholipid complexes, which increase and improve not only its stability, but also its biodistribution and absorption [46]. With these new strategies, an enhanced bioavailability and efficacy have been documented in different experimental models. Therefore, further exploration to increase the medical value for human applications are needed.

Our study was carried out on a single cell-type model. Even if preliminary studies in animals and patients are promising and coherent with our data, we should consider the possibility that similar results cannot be obtained in vivo. Moreover, since we used ultra-pure curcumin, we should warn about the risk of translating our results to commercially-available preparations of curcuma, which may contain several other compounds with contrasting effects on mucosal immunity [47]. 

## 5. Conclusions

Despite being encouraging, our results need further and deeper investigations aimed at establishing whether curcumin could be a potential therapeutic molecule for IBD and, in particular, for UC. 

In conclusion, even though our results are preliminary and need to be verified in order to be translated into the model of IBD patients, they should be considered as a first step to better characterize turmeric anti-inflammatory properties. 

## References

[1] Abraham C., Cho J.H. (2009). Inflammatory bowel disease. N. Engl. J. Med..

[2] Yang Y., Owyang C., Wu G.D. (2016). East Meets West: The Increasing Incidence of Inflammatory Bowel Disease in Asia as a Paradigm for Environmental Effects on the Pathogenesis of Immune-Mediated Disease. Gastroenterology.

[3] Benchimol E.I., Mack D.R., Guttmann A., Nguyen G.C., To T., Mojaverian N., Quach P., Manuel D.G. (2015). Inflammatory bowel disease in immigrants to Canada and their children: A population-based cohort study. Am. J. Gastroenterol..

[4] Kim H. (2012). Natural products to improve quality of life targeting for colon drug delivery. Curr. Drug Deliv..

[5] Zachos M., Tondeur M., Griffiths A.M. (2007). Enteral nutritional therapy for induction of remission in Crohn’s disease. Cochrane Database Syst. Rev..

[6] Lewis J.D., Abreu M.T. (2016). Diet as a Trigger or Therapy for Inflammatory Bowel Diseases. Gastroenterology.

[7] Vecchi Brumatti L., Marcuzzi A., Tricarico P.M., Zanin V., Girardelli M., Bianco A.M. (2014). Curcumin and inflammatory bowel disease: Potential and limits of innovative treatments. Molecules.

[8] Shishodia S. (2013). Molecular mechanisms of curcumin action: Gene expression. Biofactors.

[9] Taylor R.A., Leonard M.C. (2011). Curcumin for inflammatory bowel disease: A review of human studies. Altern. Med. Rev..

[10] Lang A., Salomon N., Wu J.C., Kopylov U., Lahat A., Har-Noy O., Ching J.Y., Cheong P.K., Avidan B., Gamus D. (2015). Curcumin in Combination With Mesalamine Induces Remission in Patients With Mild-to-Moderate Ulcerative Colitis in a Randomized Controlled Trial. Clin. Gastroenterol. Hepatol..

[11] Panahi Y., Darvishi B., Ghanei M., Jowzi N., Beiraghdar F., Varnamkhasti B.S. (2016). Molecular mechanisms of curcumins suppressing effects on tumorigenesis, angiogenesis and metastasis, focusing on NF-κB pathway. Cytokine Growth Factor Rev..

[12] Midura-Kiela M.T., Radhakrishnan V.M., Larmonier C.B., Laubitz D., Ghishan F.K., Kiela P.R. (2012). Curcumin inhibits interferon-γ signaling in colonic epithelial cells. Am. J. Physiol. Gastrointest. Liver Physiol..

[13] Jian Y.T., Mai G.F., Wang J.D., Zhang Y.L., Luo R.C., Fang Y.X. (2005). Preventive and therapeutic effects of NF-kappaB inhibitor curcumin in rats colitis induced by trinitrobenzene sulfonic acid. World J. Gastroenterol..

[14] Deguchi Y., Andoh A., Inatomi O., Yagi Y., Bamba S., Araki Y., Hata K., Tsujikawa T., Fujiyama Y. (2007). Curcumin prevents the development of dextran sulfate Sodium (DSS)-induced experimental colitis. Dig. Dis. Sci..

[15] Arafa H.M., Hemeida R.A., El-Bahrawy A.I., Hamada F.M. (2009). Prophylactic role of curcumin in dextran sulfate sodium (DSS)-induced ulcerative colitis murine model. Food Chem. Toxicol..

[16] Mastropietro G., Tiscornia I., Perelmuter K., Astrada S., Bollati-Fogolín M. (2015). HT-29 and Caco-2 reporter cell lines for functional studies of nuclear factor kappa B activation. Mediat. Inflamm..

[17] Guo Y., Shu L., Zhang C., Su Z.Y., Kong A.N. (2015). Curcumin inhibits anchorage-independent growth of HT29 human colon cancer cells by targeting epigenetic restoration of the tumor suppressor gene DLEC1. Biochem. Pharmacol..

[18] Iwamoto M., Koji T., Makiyama K., Kobayashi N., Nakane P.K. (1996). Apoptosis of crypt epithelial cells in ulcerative colitis. J. Pathol..

[19] Hagiwara C., Tanaka M., Kudo H. (2002). Increase in colorectal epithelial apoptotic cells in patients with ulcerative colitis ultimately requiring surgery. J. Gastroenterol. Hepatol..

[20] Di Sabatino A., Ciccocioppo R., Luinetti O., Ricevuti L., Morera R., Cifone M.G., Solcia E., Corazza G.R. (2003). Increased enterocyte apoptosis in inflamed areas of Crohn’s disease. Dis. Colon. Rectum..

[21] Zeissig S., Bojarski C., Buergel N., Mankertz J., Zeitz M., Fromm M., Schulzke J.D. (2004). Downregulation of epithelial apoptosis and barrier repair in active Crohn’ s disease by tumour necrosis factor alpha antibody treatment. Gut.

[22] Banerjee S., Singh S.K., Chowdhury I., Lillard J.W., Singh R. (2017). Combinatorial effect of curcumin with docetaxel modulates apoptotic and cell survival molecules in prostate cancer. Front. Biosci. (Elite Ed.).

[23] Park S.I., Lee E.H., Kim S.R., Jang Y.P. (2017). Anti-apoptotic effects of *Curcuma longa* L. extract and its curcuminoids against blue light-induced cytotoxicity in A2E-laden human retinal pigment epithelial cells. J. Pharm. Pharmacol..

[24] Zheng L., Li Y., Li X., Kou J., Zhong Z., Jiang Y., Liu Z., Tian Y., Yang L. (2016). Combination of Hydroxyl Acetylated Curcumin and Ultrasound Induces Macrophage Autophagy with Anti-Apoptotic and Anti-Lipid Aggregation Effects. Cell Physiol. Biochem..

[25] Baker M. (2017). Deceptive curcumin offers cautionary tale for chemists. Nature.

[26] Hackler L., Ózsvári B., Gyuris M., Sipos P., Fábián G., Molnár E., Marton A., Faragó N., Mihály J., Nagy L.I. (2016). The Curcumin Analog C-150, Influencing NF-κB, UPR and Akt/Notch Pathways Has Potent Anticancer Activity in Vitro and in Vivo. PLoS ONE.

[27] Li W., Wang H., Kuang C.Y., Zhu J.K., Yu Y., Qin Z.X., Liu J., Huang L. (2012). An essential role for the Id1/PI3K/Akt/NFkB/survivin signalling pathway in promoting the proliferation of endothelial progenitor cells in vitro. Mol. Cell Biochem..

[28] Cimbro R., Vassena L., Arthos J., Cicala C., Kehrl J.H., Park C., Sereti I., Lederman M.M., Fauci A.S., Lusso P. (2012). IL-7 induces expression and activation of integrin α4β7 promoting naive T-cell homing to the intestinal mucosa. Blood.

[29] Zhang W., Du J.Y., Yu Q., Jin J.O. (2015). Interleukin-7 produced by intestinal epithelial cells in response to Citrobacter rodentium infection plays a major role in innate immunity against this pathogen. Infect. Immun..

[30] Shalapour S., Deiser K., Sercan O., Tuckermann J., Minnich K., Willimsky G., Blankenstein T., Hämmerling G.J., Arnold B., Schüler T. (2010). Commensal microflora and interferon-gamma promote steady-state interleukin-7 production in vivo. Eur. J. Immunol..

[31] Shalapour S., Deiser K., Kühl A.A., Glauben R., Krug S.M., Fischer A., Sercan O., Chappaz S., Bereswill S., Heimesaat M.M. (2012). Interleukin-7 links T lymphocyte and intestinal epithelial cell homeostasis. PLoS ONE.

[32] Kanai T., Nemoto Y., Kamada N., Totsuka T., Hisamatsu T., Watanabe M., Hibi T. (2009). Homeostatic (IL-7) and effector (IL-17) cytokines as distinct but complementary target for an optimal therapeutic strategy in inflammatory bowel disease. Curr. Opin. Gastroenterol..

[33] Watanabe M., Ueno Y., Yajima T., Iwao Y., Tsuchiya M., Ishikawa H., Aiso S., Hibi T., Ishii H. (1995). Interleukin 7 is produced by human intestinal epithelial cells and regulates the proliferation of intestinal mucosal lymphocytes. J. Clin. Investig..

[34] Andreu-Ballester J.C., Pérez-Griera J., Garcia-Ballesteros C., Amigo V., Catalán-Serra I., Monforte-Albalat A., Bixquert-Jiménez M., Ballester F. (2013). Deficit of interleukin-7 in serum of patients with Crohn’s disease. Inflamm. Bowel Dis..

[35] Totsuka T., Kanai T., Nemoto Y., Makita S., Okamoto R., Tsuchiya K., Watanabe M. (2007). IL-7 is essential for the development and the persistence of chronic colitis. J. Immunol..

[36] Nemoto Y., Kanai T., Takahara M., Oshima S., Nakamura T., Okamoto R., Tsuchiya K., Watanabe M. (2013). Bone marrow-mesenchymal stem cells are a major source of interleukin-7 and sustain colitis by forming the niche for colitogenic CD4 memory T cells. Gut.

[37] Willis C.R., Seamons A., Maxwell J., Treuting P.M., Nelson L., Chen G., Phelps S., Smith C.L., Brabb T., Iritani B.M. (2012). Interleukin-7 receptor blockade suppresses adaptive and innate inflammatory responses in experimental colitis. J. Inflamm. (Lond.).

[38] Yang C., Ma X., Wang Z., Zeng X., Hu Z., Ye Z., Shen G. (2017). Curcumin induces apoptosis and protective autophagy in castration-resistant prostate cancer cells through iron chelation. Drug Des. Dev. Ther..

[39] Du X.X., Xu H.M., Jiang H., Song N., Wang J., Xie J.X. (2012). Curcumin protects nigral dopaminergic neurons by iron-chelation in the 6-hydroxydopamine rat model of Parkinson’ s disease. Neurosci. Bull..

[40] Dairam A., Fogel R., Daya S., Limson J.L. (2008). Antioxidant and iron-binding properties of curcumin, capsaicin, and S-allylcysteine reduce oxidative stress in rat brain homogenate. J. Agric. Food Chem..

[41] Galleggiante V., De Santis S., Cavalcanti E., Scarano A., De Benedictis M., Serino G., Caruso M.L., Mastronardi M., Pinto A., Campiglia P. (2017). Dendritic Cells Modulate Iron Homeostasis and Inflammatory Abilities Following Quercetin Exposure. Curr. Pharm. Des..

[42] Jiang A.J., Jiang G., Li L.T., Zheng J.N. (2015). Curcumin induces apoptosis through mitochondrial pathway and caspases activation in human melanoma cells. Mol. Biol. Rep..

[43] Sugimoto K., Hanai H., Tozawa K., Aoshi T., Uchijima M., Nagata T., Koide Y. (2002). Curcumin prevents and ameliorates trinitrobenzene sulfonic acid-induced colitis in mice. Gastroenterology.

[44] Hanai H., Iida T., Takeuchi K., Watanabe F., Maruyama Y., Andoh A., Tsujikawa T., Fujiyama Y., Mitsuyama K., Sata M. (2006). Curcumin maintenance therapy for ulcerative colitis: Randomized, multicenter, double-blind, placebo-controlled trial. Clin. Gastroenterol. Hepatol..

[45] Anand P., Kunnumakkara A.B., Newman R.A., Aggarwal B.B. (2007). Bioavailability of curcumin: Problems and promises. Mol. Pharm..

[46] Prasad S., Tyagi A.K., Aggarwal B.B. (2014). Recent developments in delivery, bioavailability, absorption and metabolism of curcumin: The golden pigment from golden spice. Cancer Res. Treat..

[47] Holt P.R. (2016). Curcumin for Inflammatory Bowel Disease: A Caution. Clin. Gastroenterol. Hepatol..

